# From *Drosophila* to Human: Biological Function of E3 Ligase Godzilla and Its Role in Disease

**DOI:** 10.3390/cells11030380

**Published:** 2022-01-23

**Authors:** Valérie C. Cabana, Marc P. Lussier

**Affiliations:** 1 Département de Chimie, Université du Québec à Montréal, Montréal, QC H2X 2J6, Canada; cabana.valerie@courrier.uqam.ca; 2 Centre d’Excellence en Recherche sur les Maladies Orphelines—Fondation Courtois (CERMO-FC), Faculté des Sciences, Université du Québec à Montréal, Montréal, QC H2X 3Y7, Canada

**Keywords:** ubiquitin, Godzilla, RNF13, RNF167, ZNRF4, pathological dysfunction, E3 ligase, PA-TM-RING family

## Abstract

The ubiquitin–proteasome system is of fundamental importance in all fields of biology due to its impact on proteostasis and in regulating cellular processes. Ubiquitination, a type of protein post-translational modification, involves complex enzymatic machinery, such as E3 ubiquitin ligases. The E3 ligases regulate the covalent attachment of ubiquitin to a target protein and are involved in various cellular mechanisms, including the cell cycle, cell division, endoplasmic reticulum stress, and neurotransmission. Because the E3 ligases regulate so many physiological events, they are also associated with pathologic conditions, such as cancer, neurological disorders, and immune-related diseases. This review focuses specifically on the protease-associated transmembrane-containing the Really Interesting New Gene (RING) subset of E3 ligases. We describe the structure, partners, and physiological functions of the *Drosophila* Godzilla E3 ligase and its human homologues, RNF13, RNF167, and ZNRF4. Also, we summarize the information that has emerged during the last decade regarding the association of these E3 ligases with pathophysiological conditions, such as cancer, asthma, and rare genetic disorders. We conclude by highlighting the limitations of the current knowledge and pinpointing the unresolved questions relevant to RNF13, RNF167, and ZNRF4 ubiquitin ligases.

## 1. Introduction

Protein post-translational modification (PTM) refers to a modification of a polypeptide chain that occurs after its biosynthesis. These protein alterations typically require enzymes that catalyze either reversible or irreversible biochemical modifications and, thus, add another level of complexity and regulation to the proteome. It is clear from a vast array of studies that PTMs play crucial roles in influencing protein activity and, consequently, regulate the cellular functions and signaling pathways [1]. The array of PTMs includes chemical modifications, such as phosphorylation, modification by complex molecules, such as glycosylation, or peptide modification, such as ubiquitination [2].

**Figure 1:**
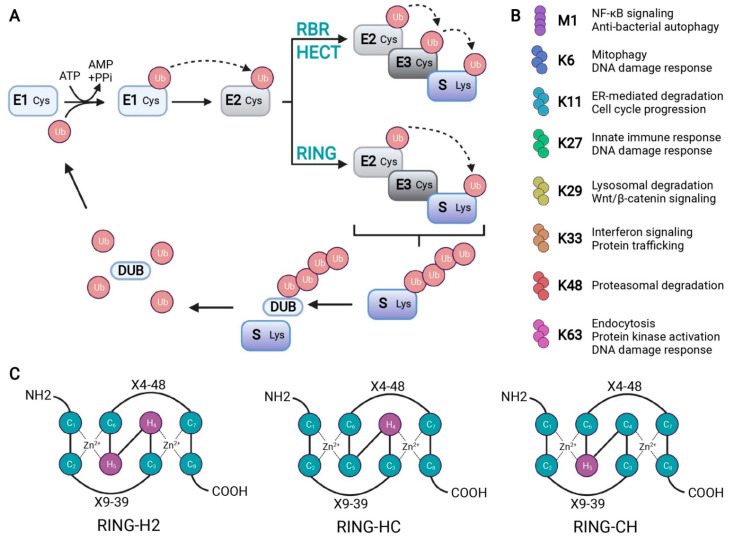
Schematic representation of the ubiquitination process. (**A**) Ubiquitination starts with an ATP-dependent step where the ubiquitin-activating enzyme (E1) creates a thioester bond between the cysteine residue at its active site and ubiquitin (Ub). Ub is then transferred to the cysteine residue in the Ub-conjugating enzyme (E2) active site. Finally, Ub ligase (E3) transfers Ub to a lysine residue of the substrate (S). There are three types of E3s, as follows: RBR, HECT, and RING domains. RBR and HECT domain E3 ligases first transfer Ub to a cysteine residue of their active site before transferring it to the substrate. The RING domain directly transfers Ub from E2 to the substrate. Deubiquitinases (DUB) can remove, edit the length of, or disassemble a Ub chain to be recycled. (**B**) A description of the cellular function for the possible Ub chains formed through methionine (M)1 or lysine (K) 6, 11, 27, 29, 33, 48, or 63. (**C**) Schematic representation of the cross-brace arrangements of the RING-H2, RING-HC, and RING-CH finger motifs. Cysteine (C) and histidine (H) residues are numbered with their positions in the conserved zinc- (Zn^2+^-) coordination sites. X represents any amino acid located in the polypeptide between the cysteines coordinating Zn^2+^.

Ubiquitination is a reversible PTM that involves the covalent addition of one or more ubiquitin (Ub) molecules, a 76-amino acid protein, on a substrate’s internal lysine via the formation of an isopeptide bond. The ultimate conjugation of Ub to a substrate requires the sequential action of a Ub-activating enzyme (E1), a Ub-conjugating enzyme (E2), and a Ub ligase enzyme (E3) [3,4]. The E1 generates a thioester bond between the cysteine at its active site and the Ub *C*-terminal carboxyl group in an ATP-dependent process [4,5,6]. Through a transesterification reaction, the E2 catalyzes the transfer of Ub from the E1 to the cysteine residue in the active site of the E2 to form a Ub~E2 conjugate [4,5,6]. Finally, the E3 promotes the discharge of Ub from the Ub~E2 conjugate to promote the formation of an isopeptide bond between the acceptor lysine of the target protein and the *C*-terminal glycine of Ub [4,5,6] (Figure 1A). Ub contains seven lysine residues (K6, K11, K27, K29, K33, K48, and K63) and one methionine residue (M1), all of which can be targeted for Ub coupling, leading to distinct poly-Ub chains and an expanded “ubiquitin code” [3,7,8,9,10,11,12,13,14] (Figure 1B). For instance, whereas mono-ubiquitination is usually involved in endocytosis, DNA repair, and signal transduction, poly-ubiquitination is extremely diverse and is used for a variety of signals [15,16]. For example, the K48 chains are involved in proteasomal degradation, while the K63 chains are implicated in proteasome-independent signaling, such as endocytosis, protein kinase activation, and the DNA damage response [7,17,18]. Furthermore, the K29 chains are involved in lysosomal degradation, while the K11 chains are used to regulate endoplasmic reticulum-mediated degradation and cell cycle progression [13,14,19,20,21]. Importantly, Ub chain linkages may need editing and removal from the substrate proteins, a process requiring enzymes called deubiquitinases (DUBs) that exhibit specificity toward the different Ub chain linkage topologies and structures [4,6].

Deciphering the Ub code and its associated mechanisms is central to understanding the regulation of protein activity, cellular signaling, and cell physiology. One of the major lines of investigation that has motivated the Ub research community over the last decade is to uncover how genetically inherited errors can lead to devastating diseases. For instance, important advances have demonstrated genetic links in Angelman syndrome (caused by maternal deficiency of the E6-AP Ub E3 ligase), juvenile recessive Parkinson’s disease (caused by mutations in the gene encoding Parkin-Ub E3 ligase), and autoimmune polyendocrine syndrome type I (caused by mutations in the gene encoding the AIRE Ub E3 ligase) [22]. The E3 ligases are a distinct group of enzymes that are crucial to the ubiquitination process as they control the specificity of the reaction towards a targeted protein.

To date, more than 600 genes encoding E3 ligases have been identified in the human genome [3,4,8]. Based on their catalytic core domain, E3 ligases are architecturally diverse and are categorized according to their functional domain consisting of either a Homologous to E6-AP C-Terminal (HECT) domain, a Really Interesting New Gene (RING) domain, or a RING-Between-RING (RBR) domain [6] (Figure 1A). Specifically, the HECT-type E3s catalyze Ub transfer from Ub~E2 to a substrate via their active-site cysteine residue [4,5,6]. In contrast, a RING-type E3 does not possess an active cysteine in its functional RING domain and, thus, mediates the transfer of Ub to the substrate protein from the Ub-charged E2. Importantly, this family harbors a zinc-binding domain termed the RING, or the U-box domain, as the platform required to associate with Ub~E2s. Finally, RBR-type E3s are considered to be a hybrid of the HECT and RING domains since these enzymes function by using an E2-binding RING structure and a second RING domain that contains an active cysteine for the formation of an intermediate E3~Ub conjugate before the transfer of Ub to a substrate [6,23].

While there are about 30 HECT domain E3 proteins in mammals, there are more than 300 potential RING finger-encoding proteins in the human genome [24,25]. Of those, about 50 are predicted to have hydrophobic regions corresponding to transmembrane (TM) domains [24,26]. The TM-containing RING finger proteins can be further divided into subfamilies that include the tripartite motif-containing (TRIM), the protease-associated (PA) transmembrane RING (PA-TM-RING), the RBR, and the membrane-associated RING-CH (MARCH) proteins. Except for the presence of a RING finger domain, these four transmembrane RING finger-containing protein families share little sequence homology and have diverse subcellular localizations and functions [26].

The presence of an N-terminal putative signal peptide (SP), a PA domain, a single TM domain, and a RING-H2 finger domain characterize the PA-TM-RING family [26,27]. The signal peptide allows the targeting of the protein to the secretory pathway. This region of the protein has yet to be adequately studied for most, if not all, of the members of this family [28]. The PA domain is a conserved sequence of about 120 amino acids implicated in protein-protein interactions [27,29]. It was initially identified in non-catalytic regions of plant vacuolar sorting receptors and zinc-containing metalloproteases, but the exact role of this domain in the PA-TM-RING family of Ub ligases remains unclear [27,30]. The single TM domain is composed predominantly of nonpolar amino acid residues and is required for anchoring the protein in the membrane [31]. The RING finger domain is divided into subgroups based on the presence of cysteine (C) and histidine (H) residues in the fourth and fifth positions of the catalytic domain [26]. The RING-HC domain is composed of a C_3_HC_4_, the RING-H2 has a C_3_H_2_C_3_ sequence, and the RING-CH contains a C_4_HC_3_ [26,32] (Figure 1C). The PA-TM-RING family has a RING-H2 finger domain with two characteristic histidines in the fourth and fifth positions of the conserved Zn^2+^-coordination sites [26,32].

**Figure 2:**
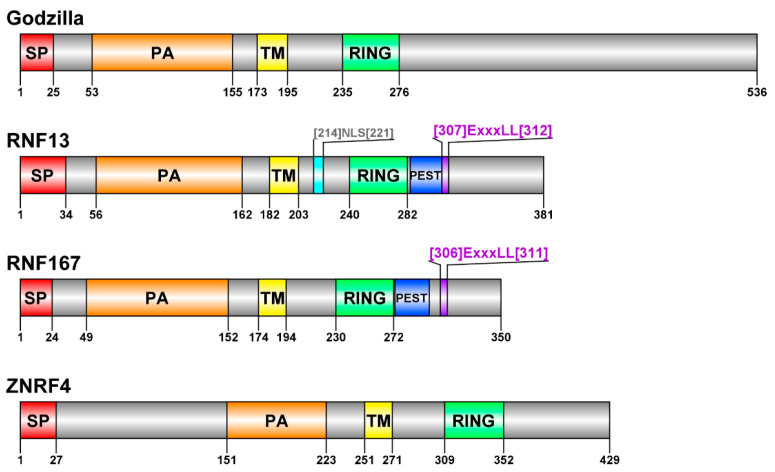
Schematic domain structure of *Drosophila* Godzilla, human RNF13, human RNF167, and human ZNRF4 proteins. SP, signal peptide (in red); PA, protease-associated domain (in orange); TM, transmembrane domain (in yellow); NLS, nuclear localization signal (in light blue); RING, RING domain (in green); PEST, PEST domain (in deep blue); ExxxLL, dileucine motif (in purple). Graphical representations were generated with DOG 2.0.1: Illustrator of Protein Domain Structures [37].

The PA-TM-RING family is present in *Drosophila melanogaster* where it is known as the Goliath Ub Ligase family, with only two members, Goliath and Godzilla. While these two are the only representatives of the PA-TM-RING family in *Drosophila melanogaster*, there are nine predicted homologues in humans. RNF133, RNF148, RNF128/GRAIL, RNF130, RNF150, and RNF149 are closely related to Goliath whereas RNF167, RNF13, and ZNRF4 are homologs of Godzilla [33] (Figure 2). The *Goliath* gene was first identified in 1987 and was shown to be a transcription factor involved in *Drosophila* embryo mesoderm formation [34,35]. It was later demonstrated to be enriched after Notch activation and was predicted to be active in fusion-competent myoblasts [36]. Since the *Goliath* gene expression is restricted to embryonic muscle, the *Drosophila Goliath* null mutants are viable and fertile [33,36].

While much is known about the human Goliath homologues, such as the role of RNF128 in immune tolerance [38,39,40], this review will exclusively focus on Godzilla and its human homologues. First, we will address the Godzilla function in *Drosophila* and then present the cellular localization, function, and pathological dysfunction for Godzilla’s human homologues, RNF167, RNF13, and ZNRF4. For instance, hundreds of single-nucleotide variation (SNV) mutations for RNF167, RNF13, and ZNRF4 have been reported in cancer. Finally, we will highlight some of the remaining questions about these proteins.

## 2. Godzilla

The *Godzilla* gene was first discovered in 2000 when Adams et al. determined the nucleotide sequence of nearly all of the euchromatic portion of the *Drosophila* genome [41]. Godzilla is universally expressed in *Drosophila*, and alterations to this locus are larval lethal [33]. The Godzilla protein sequence includes the characteristic domains of the family (Figure 2). When exogenously expressed in mammalian human embryonic kidney 293 (HEK293T) cells, Godzilla significantly co-localized with the early endosomal marker Early Endosome Antigen 1 (EEA1). This is concordant with the observations of endogenous Godzilla protein overlapping with endosomes in *Drosophila* tissues [33]. Furthermore, exogenous expression of Godzilla resulted in enlarged endosomes and a loss of Mannose-6-Phosphate (M6P) receptor staining, suggesting that the Godzilla expression could perturb the endosomal maturation process [33]. However, the use of the Godzilla Ub ligase-dead mutant (DN for dominant-negative) did not result in enlarged endosomes, implying that Godzilla’s impact on endosomal maturation is through RING-dependent substrate ubiquitination [33].

### 2.1. Godzilla’s Substrate Regulates Recycling Endosomes

The vesicle-associated membrane protein 3 (VAMP3), a member of the Soluble N-ethylmaleimide-sensitive-factor Attachment protein Receptor (SNARE) family, is a substrate for Godzilla [33]. Specifically, the enlarged endosome phenotype observed with Godzilla overexpression in HEK293T cells was blocked both by abrogating the VAMP3 activity via small interfering RNA (siRNA) and by the use of VAMP3 mutants that could not be ubiquitinated in HEK293T cells, implicating VAMP3 ubiquitination by Godzilla in the regulation of SNARE-dependent fusion [33]. Knowing that VAMP3 is involved in the exocytosis of recycling endosomes [42,43], the presence of Rab11-positive recycling vesicles, which normally exhibit a pericentriolar location, was analyzed in Godzilla-transfected cells [33]. Wild-type (WT) Godzilla-expressing cells showed residual Rab11 staining in the vicinity of the enlarged endosomes, meaning that the Rab11-containing vesicles were unable to properly form in the presence of Godzilla [33]. Interestingly, the DN Godzilla did not induce the same phenotype, suggesting that the impact on recycling endosomes is through RING-dependent ubiquitination [33]. Furthermore, transferrin internalization was delayed in the cells expressing the active form of Godzilla, in contrast to the DN Godzilla, further supporting the role of Godzilla in the regulation of recycling endosome trafficking [33]. Importantly, the knockdown of the *Drosophila* homologue of VAMP3, Synaptobrevin (Syb), in *Drosophila* wing disc led to results similar to those described above, confirming the role of Godzilla in vivo [33].

### 2.2. Wingless Transcytosis

Usually, to prevent extracellular protein transfer, the apical and basolateral membranes of epithelia are insulated from each other [44]. Transcytosis is the vesicular transport process that allows proteins to cross the cellular barriers [44]. Interestingly, a role for Godzilla was uncovered in Wingless transcytosis where Godzilla promotes signaling in *Drosophila* wing imaginal discs [45]. Yamazaki et al. have found that Wingless is first produced apically before being transcytosed to the basolateral surface, where it engages in a signaling pathway [45]. The authors demonstrated that this transcytosis is dependent on Godzilla [45]. Godzilla knockdown led to the loss of the wing margin, a reduced expression of Senseless, which is a target of Wingless signaling, and the accumulation of intracellular Wingless in the cellular apical region [45]. In agreement with the preceding demonstration that Godzilla ubiquitinates Syb [33], Syb mutants and Syb knockdown led to a similar phenotype as the one observed in the Godzilla knockdown cells [45]. Overall, these studies suggest that Godzilla has important roles in regulating recycling endosome trafficking and in Wingless transcytosis through the ubiquitination of the Syb protein [33,45].

## 3. RNF13

Ring Finger Protein 13 (RNF13) was first identified in 1996 as chicken RING zinc finger (C-RZF) when Tranque et al. studied the changes in gene expression following tenascin-C treatment in chicken embryo brains [46]. They found that the C-RZF gene expression was upregulated, but the implication of this effect is still unknown [46]. The RNF13 sequence includes the characteristic domains of the PA-TM-RING family, in addition to a PEST domain (described below), a nuclear localization signal (NLS), and a canonical [D/E]xxxL[L/I] motif (Figure 2) [47]. Until now, the mammalian RNF13 protein mostly has been localized to the endoplasmic reticulum (ER), Golgi, late endosomes, lysosomes, and the plasma membrane [27,47,48,49,50].

### 3.1. Protein Domains

#### 3.1.1. PEST Domain

An intriguing region within RNF13 is the PEST domain, which contains a high number of proline (P), glutamic acid (E), serine (S), and threonine (T) residues usually, but not always, with clusters of several positively charged amino acids that promote the rapid degradation of short half-life proteins [51]. The short half-life of RNF13 was hypothesized to be associated with its PEST domain. Specifically, it was demonstrated that the exogenous expression of RNF13 in Chinese hamster ovary (CHO) cells was barely detectable unless the cells were treated with the proteasomal inhibitor MG132 [47,50]. Interestingly, RNF13 abundance was stabilized in the cells treated with the protein kinase C (PKC) agonist phorbol 12-myristate 13-acetate (PMA), increasing RNF13’s half-life similar to that of MG132 [50]. However, to the best of our knowledge, no study has evaluated the implication of the PEST domain in RNF13 protein turnover and, specifically, whether MG132 or PMA treatment act through this domain to increase RNF13’s half-life.

#### 3.1.2. Nuclear Localization Signal

RNF13 appears to possess an NLS, but with an undetermined function. The treatment of HeLa cells with PMA caused RNF13 to be targeted to the inner nuclear membrane (INM) [50], suggesting a potential nuclear role for the protein in response to some cellular signals [50]. Mechanistically, RNF13 targeting to the INM requires the protein to first traffic through the endosomes [50]. RNF13 undergoes extensive proteolysis in order to release both the PA- and the RING-domains from the membrane [47]. However, the role of RNF13 in the nucleus, and why it undergoes such proteolytic events, remain to be elucidated.

#### 3.1.3. Di-Leucine Sorting Signal

Another region of interest in the RNF13 protein sequence is the presence of a canonical [D/E]xxxL[L/I]-type di-leucine sorting signal. This region binds to adaptor protein (AP) complexes to allow proper sorting, targeting, and routing of a transmembrane protein, such as RNF13, within the secretory pathway and endolysosomal compartments [52,53]. Recent studies using affinity-purification and mass spectrometry analyses [54,55,56] have identified an interaction between RNF13 and AP-1, an AP complex associated with the trans-Golgi network, and with early and recycling endosomes [57,58]. It was also found that RNF13 associates with AP-3 [54,55,56], which mediates the transport from the tubular endosomes to the late endosomes and lysosomes and is therefore involved in the biogenesis of lysosome-related organelles [57,58]. Accordingly, our own work demonstrates that RNF13 interacts with the AP-3 complex in HEK293T cells [59], an interaction disrupted by an AP-binding defective RNF13 mutant. By replacing the leucines with alanines in the di-leucine motif (i.e., L311A/L312A), the lysosomal localization of RNF13 was altered, suggesting that RNF13′s di-leucine sorting signal motif is crucial for the lysosomal targeting of the protein [59]. Correspondingly, the knockdown of AP-3 altered the lysosomal localization of WT RNF13 and led to an alteration in the endosomal vesicle size [59]. Overall, this suggests that RNF13 might go into the endolysosomal compartments through an AP-3 dependent pathway. Future studies are critical to clarify the mechanism leading to the endolysosomal localization of RNF13 [59].

### 3.2. Cellular Functions

#### 3.2.1. Neurobiological Role

Over the years, RNF13 expression has been associated with neurobiological functions. For instance, the ectopic expression of RNF13 allows the spontaneous growth of neurites in PC12 cells, which is similar to the nerve growth factor treatment of these cells [60]. In addition, RNF13 mRNA expression doubled in the B35 cells when they were treated with dibutyryl-cAMP [47]. Together, these studies implicate RNF13 in neurodevelopment. Accordingly, evidence shows that RNF13 gene expression in humans is ubiquitous, but higher in the brain, cerebellum, spinal cord, and testis [47,60,61]. Interestingly, a study using RNF13 knockout mice displaying a deficit in learning showed that the synaptic vesicle density decreased while the active zone size increased, suggesting that these observations resulted from the impaired SNARE complex assembly [62]. The study also demonstrates that RNF13 ubiquitinates SNARE-associated protein (snapin) to allow its interaction with Synaptosome associated protein 25 (SNAP-25) to strengthen the SNARE complex [62]. Co-immunoprecipitation assays demonstrated that RNF13 knockout mice have decreased interaction between snapin/SNAP-25, Vamp-2/SNAP-25, Synaptotagmin/SNAP-25, and Synaptotagmin/Vamp-2, suggesting that RNF13 plays a critical role in synaptic neurotransmission [62]. Interestingly, while the authors reported K29-linked polyubiquitination of snapin by RNF13, increasing snapin protein expression was correlated in a linear relationship with higher RNF13 protein expression, suggesting that RNF13 does not promote snapin degradation [62]. While K29 polyubiquitination has been reported to regulate some kinase activities, the role for RNF13-mediated snapin K29-linked Ub chains remains to be elucidated [63].

#### 3.2.2. Cell Migration and Invasiveness

In addition to its implication in neurobiology, RNF13 has been associated with tumorigenesis, ER stress, and myogenesis [48,49,64,65]. In pancreatic cancer, the overexpression of WT, but not DN, RNF13 in MiaPaca-2 cells led to increased matrix metallopeptidase 9 (MMP-9) activity, suggesting that the Ub ligase activity of RNF13 promotes metalloprotease function and cell invasion [49]. In contrast, RNF13 knockout mice implanted with a pulmonary metastatic cancer cell model did not have a difference in tumor size when compared to their WT littermates, but the WTs exhibited a larger metastatic area in the lung [66]. Interestingly, this study shows that RNF13 knockout mice had a reduced granulocyte macrophage colony-stimulating factor (GM-CSF) concentration in the lung, suggesting that RNF13 reduces the colonization of metastases through the regulation of GM-CSF concentration [66]. Whereas the molecular mechanism of RNF13 regulation of GM-CSF concentration is not yet understood, it is well known that invasion is the first step to metastasis formation while the host microenvironment is key to establishing the final fate of the tumor cells [66]. Thus, it is possible that a higher expression of RNF13 leads to enhanced invasiveness but also reduces the likelihood of metastatic colonization. In order to shed light on the discrepancy of these two studies [49,66], future studies will need to dissociate the function of RNF13 at the protein network level (i.e., with various interactors) and the Ub ligase activity.

#### 3.2.3. ER Stress

An important implication in cellular apoptosis has been reported for RNF13. Specifically, the ectopic expression of RNF13 increases apoptosis while RNF13 knockdown led to cell resistance to apoptosis, induced by treatment using the PKC inhibitor staurosporine (STS) [48]. Specifically, RNF13 ectopic expression in SHSY-5Y cells increased the amount of spliced X-Box Binding Protein 1 (XBP1), a result similar to that of ER stress induction using tunicamycin [48]. Conversely, RNF13 knockdown reduced both the amount of spliced XBP1, phosphorylated c-Jun N-terminal kinase (JNK), and phosphorylated c-Jun in cells treated with tunicamycin [48]. The authors also demonstrated that RNF13 interacts with inositol-requiring enzyme 1α (IRE1α). The ectopic expression of RNF13 increases the IRE1α phosphorylation, suggesting that RNF13 regulates ER stress-induced apoptosis through the activation of the IRE1α-TNF receptor-associated factor 2 (TRAF2)-JNK signaling pathway [48]. Supporting this idea, a cycloheximide chase experiment demonstrated that the RNF13–IRE1α interaction increased the half-life and the stability of IRE1α [67].

#### 3.2.4. Myogenesis and Muscle Regeneration

The role of RNF13 in myogenesis has only been studied in chickens and mice [65,68]. Specifically, myostatin, a negative regulator that inhibits myoblast proliferation and differentiation, induces C-RZF gene expression in chicken fetal myoblasts (CFM) [65]. The analysis of the temporal pattern of the C-RZF protein has revealed that it was highly expressed in embryonic skeletal muscle, but rapidly decreased to almost undetectable levels at one week after hatching [65]. Furthermore, ectopic expression of C-RZF in CFM led to a reduced cell number. An important experiment using [^3^H]-thymidine incorporation suggested that the decreased cell number was not a result of increased cell death, but more likely an inhibitory effect of C-RZF on CFM proliferation [65]. Following this idea, the authors looked at the expression of MyoD and Caveolin-3, two myogenic regulatory factors. They found that both MyoD and Caveolin-3 were downregulated in C-RZF-overexpressing CFM, suggesting that C-RZF could act as a negative regulator of muscle cell proliferation. To our knowledge, whether human RNF13 has a similar function remains to be seen [65].

Another study has investigated the function of RNF13 in cardiotoxin (CTX)-induced skeletal muscle regeneration. The results show that after CTX damage, RNF13 knockout mice had more regenerating fibers in their tibialis anterior (TA) muscles than their WT littermates [68]. Likewise, satellite cell proliferation was also accelerated while cytokines interleukin-4 (IL-4) and interleukin-6 (IL-6) were upregulated after muscle damage in the CTX-treated RNF13 knockout mice [68]. Although the study did not explain how RNF13 acts on these cytokines, the use of neutralizing antibodies against those two cytokines slowed the regeneration process in the CTX-treated RNF13 knockout mice, suggesting that the effect of RNF13 on cell proliferation involves a tight regulation of IL-4 and IL-6 levels [68].

### 3.3. Pathological Dysfunctions Associated with RNF13

#### 3.3.1. Cancer

One study investigated some of the RNF13 variants identified in tumor samples [27]. While the overexpression of RNF13 WT proteins induced giant endosomes in HEK293T cells, only the variants located within the PA or RING domain lost the ability to induce this phenotype [27]. Interestingly, the point mutations in the RING domain allowed RNF13 to retain its endosomal localization, while the mutations in the PA domain did not [27]. Furthermore, altered RNF13 expression has been linked to cancer. Indeed, RNF13 expression is downregulated in uveal melanoma [69], while RNF13 expression was found to be significantly higher in pancreatic ductal adenocarcinoma (PDAC) than in normal, healthy pancreatic tissue [49]. The upregulated RNF13 expression was shown to be associated with histological grading, and the positive staining of RNF13 was mostly found in well-differentiated tumors [49].

#### 3.3.2. Atherosclerotic Plaques

RNF13 altered expression is not only linked to cancer. In fact, recent studies have shown that RNF13 gene expression is upregulated in atherosclerotic plaques [70] and downregulated in asthma [71]. Also, Huang et al. discovered that the microRNA miR-32-3p regulates the expression of RNF13 [72]. Specifically, the expression of miR-32-3p was downregulated in cells that were treated with oxidized low-density lipoprotein (ox-LDL) and TNF-α to simulate an ER stress model, as observed in atherosclerosis [72]. Under the same conditions, RNF13 mRNA expression and protein abundance were upregulated [72]. In addition, ectopic expression of RNF13 in the presence of ox-LDL and TNF-α led to an increase in stress-induced apoptosis [72]. Interestingly, bioinformatics analysis revealed that miR-32-3p was also downregulated in patients with acute myocardial infarction [72]. In an atherosclerotic plaque model in rats, it was found that the inhibition of miR-32-3p led to higher RNF13 expression, an upregulation of ER stress-related proteins, higher arterial plaque instability, a lower survival rate, and increased pathological lesions in the arterial tissue. These results suggest that RNF13 might be involved in atherosclerotic plaques via its role on ER stress-induced cell apoptosis [72].

#### 3.3.3. Parkinson’s Disease

A recent study demonstrated that silencing RNF13 could alleviate the symptoms in a Parkinson’s disease mouse model [73]. Specifically, the injection of a short hairpin RNA specific for RNF13 (shRNF13) with an intraperitoneal injection of 1-methyl-4-phenyl-1,2,3,6-tetrahydropyridine (MPTP) to induce the Parkinson’s disease model demonstrated that the group with the silenced RNF13 showed improved motor coordination when compared to an MPTP model only [73]. Importantly, the apoptosis of neurons in the substantia nigra was reduced in shRNF13 + MPTP when compared to MPTP alone. Interestingly, the authors measured a significant reduction in the protein abundance for the different components of the IRE1α-TRAF2-Apoptosis signal-regulating kinase 1 (ASK1)-JNK pathway [73]. Furthermore, dopaminergic neurons in the substantia nigra pars compacta had significantly more immunohistochemical staining of tyrosine hydroxylase-immunoreactive (TH-IR) in the neurons from the MPTP + shRNF13 mice when compared to MPTP alone [73]. Overall, this study suggests that reducing the activation of the IRE1α-TRAF2-ASK1-JNK pathway by silencing RNF13 might be beneficial to alleviate some of the motor dysfunction and to prevent the dopaminergic neuron damage in a Parkinson’s disease mouse model [73].

#### 3.3.4. Developmental and Epileptic Encephalopathy 73

Edvardson et al. reported three unrelated individuals with developmental and epileptic encephalopathy 73 (DEE73, OMIM #618379) carrying a de novo heterozygous mutation in the gene encoding RNF13 (c.932T>C [p.L311S] and c.935T>C [p.L312P]) [74]. These individuals had clinical features including, but not limited to, microcephaly, feeding difficulties, failure to thrive, restlessness, refractory epilepsy, cortical visual impairment, bilateral hearing loss, scoliosis, and a profound intellectual disability [74]. In this report, fibroblast and lymphoblast cells derived from an affected individual carrying the RNF13 variant L311S were treated with tunicamycin to induce ER stress [74]. The results showed that both spliced XBP1 and phosphorylated c-Jun were increased in the treated cells from the affected individual compared to an unrelated control, suggesting that the mutated cells had enhanced ER stress-induced apoptosis signaling [74]. Although this seminal study provided important evidence regarding the biological implication of expressing RNF13 variant L311S, it did not address the fact that the identified variants L311S and L312P completely altered RNF13’s [D/E]xxxL[L/I]-type di-leucine sorting signal. In our own study, we demonstrated that both RNF13 L311S and L312P variants disrupted the association with AP-3 [59]. Notably, both RNF13 L311S and L312P variants no longer localized in the lysosomes, although they changed the morphology of the endolysosomal vesicles [59]. While we don’t yet know the exact molecular mechanism involved and how it is linked to the increased apoptosis markers described by Edvardson et al., our results suggest that RNF13 L311S and L312P disrupt the endolysosomal pathway due to the loss of interaction with the AP-3 complex [59,74]. Future studies will need to pinpoint the molecular and cellular mechanisms that are altered by the presence of the RNF13 variants and to investigate the possibility that enhanced ER stress-induced apoptosis signaling may reflect an altered endolysosomal function.

## 4. RNF167

Ring Finger Protein 167 (RNF167), initially named RING105 [75], was first isolated in a bacterial two-hybrid screen with hCdc34/UbcH3 as bait [75]. The RNF167 sequence (Uniprot Accession Q9H6Y7) includes the characteristic domains of the PA-TM-RING family, along with a PEST domain (Figure 2) [75]. As for RNF13, the presence of the PEST domain in RNF167 could indicate a short half-life of the protein. Accordingly, the ectopically expressed WT RNF167, but not DN RNF167, is rapidly degraded in both HeLa and HEK293T cells in a process sensitive to the proteasome inhibitor MG132, which inhibits the degradation of the protein [75]. The experiments performed in our laboratory do not support the statement related to MG132, and the reason for this discrepancy is unclear. Similar to RNF13, RNF167 includes a canonical di-leucine motif that has never been studied. Interestingly, affinity-purification and mass spectrometry analyses [55,56] demonstrate that RNF167 interacts with AP-3, which supports the previous studies describing RNF167 localization in late endosomes and lysosomes [27,76,77,78]. Future molecular studies will likely define the importance of the di-leucine motif in RNF167 trafficking to endolysosomal compartments.

### 4.1. Expression

The RNA expression of RNF167 in various tissues has been reported in the literature and, intriguingly, is highly divergent. For instance, using qPCR on Human Total RNA Master Panel II, RNF167 expression was elevated in the brain, fetal brain, cerebellum, and testis but was low in the pancreas [61]. On the other hand, using Human MTC Panel I and II, RNF167 expression was reported to be higher in the liver, pancreas, and testis, while being lower in the brain [76]. Although both of these studies show that RNF167 is ubiquitously expressed, the divergence may be associated with the normalization of the samples [61,76]. Importantly, RNF167 protein abundance in tissue was verified and found to be higher in the kidney and liver of human tissues [75]. In the rodent brain, the cortex and hippocampus express similar levels of the RNF167 protein [76] in the detergent soluble synaptosome membrane fraction but excluded from the postsynaptic density [77].

### 4.2. Neurotransmission Modulator

In the brain, RNF167 is an important regulator of excitatory neurotransmission [76,77]. The expression of either WT or DN RNF167 in neurons (endogenous expression), and in HEK293T (ectopic expression), did not affect the total protein abundance of α-amino-3-hydroxy-5-methyl-4-isoxazole propionic acid (AMPA) receptor (AMPAR) subunits GluA1 or GluA2 [76]. However, the surface expression of GluA2, a subunit of the AMPAR that plays a major role in excitatory synaptic transmission, was highly increased in the neurons that were infected with lentiviruses expressing DN RNF167 or shRNAs designed to knockdown endogenous RNF167 [76]. The alteration of RNF167 activity led to an increase in evoked excitatory postsynaptic AMPAR currents (AMPAR-EPSCs) at the Schaffer collateral/CA1 synapses [76]. This phenotype could be rescued by RNF167 WT overexpression [76]. Overall, these results suggest that ubiquitination of GluA2 controls its surface expression and RNF167 regulates the neuronal synaptic strength [76].

To better understand the mechanisms underlying RNF167-mediated AMPAR ubiquitination and how it affects AMPAR synaptic targeting, Ghilarducci et al. characterized the interaction between Ub-conjugating E2 enzymes (E2s) and RNF167 [77]. Using in vitro ubiquitination, it was demonstrated that RNF167 could functionally interact with approximately fifteen E2s [77]. Interestingly, UBE2D1-4 and UBE2N interact with RNF167 WT with dissociation constants in the high nanomolar range [77]. Immunofluorescence microscopy confirmed that E2s and RNF167 were localized in the same intracellular compartment, and the ability of RNF167 to interact with and ubiquitinate GluA2 was demonstrated using in vitro binding and ubiquitination assays [77]. Interestingly, using a two-step in vitro ubiquitination assay, it was shown that UBE2D1 is first needed for mono-ubiquitination before the complex UBE2N/UBE2V1 can promote poly-ubiquitination of GluA2 in an RNF167-dependent manner [77]. Ultimately, the AMPA agonist-induced GluA2 ubiquitination in the hippocampal neurons was reduced in the cells that were pretreated with a UBE2N/UBE2V1 complex inhibitor, suggesting that the UBE2N/UBE2V1 enzymatic complex is involved in activity-dependent GluA2 ubiquitination [77]. Due to the fact that AMPARs are modified with a K63-Ub chain [79] and that UBE2N is responsible for assembling this chain [5], it is highly plausible, but remains to be clearly demonstrated, that RNF167 catalyzes the formation of a K63-specific polyubiquitination chain on GluA2.

### 4.3. Substrates Regulate Endosomal Trafficking and Lysosome Positioning

In addition to neuronal function, RNF167 also regulates endosomal trafficking and lysosome positioning [33,78,80]. The exogenous expression of RNF167 in HEK293T led to a phenotype similar to the overexpression of Godzilla, which caused enlarged EEA1-positive early endosomes and a loss of Rab11-positive recycling endosomes [33]. This study was the first to demonstrate the RNF167-dependent ubiquitination of Vamp3, suggesting that RNF167 regulates endosomal recycling in a manner similar to Godzilla [33]. A few years later, a proximity-dependent biotin labelling approach led to the identification of the ADP-ribosylation factor-like protein 8B (Arl8B) as a new substrate for RNF167 [78]. RNF167 targets Arl8B for degradation via the proteasome-dependent pathway in HeLa cells [78]. The level of Arl8B protein decreased when the RNF167 protein level increased, and this effect on the Arl8B protein abundance was inhibited by MG132 treatment or by the depletion of RNF167 using RNF167-specific siRNAs [78]. Knowing that Arl8B regulates lysosome localization and the knockdown of Arl8B causes the perinuclear clustering of lysosomes, the study showed that lysosomes were clustered in the perinuclear region in the presence of RNF167 WT but not DN [78]. Specifically, they demonstrated that RNF167 caused the clustering of the lysosomes in the presence of Arl8B WT, but not with the K141R mutant that could not be ubiquitinated, suggesting a role of RNF167 in lysosome positioning through Arl8B degradation [78]. Accordingly, the exogenous expression of RNF167 delays epidermal growth factor receptor (EGFR) degradation in contrast to siRNF167, which increases the degradation rate [78]. Specific siRNAs against Vamp3 and Arl8B also demonstrated that Vamp3 did not influence EGFR degradation while the knockdown of Arl8B recapitulates the RNF167 overexpression phenotype, suggesting that Arl8B degradation by RNF167 inhibits the EGFR trafficking to lysosomes [78].

Another study demonstrated that RNF167 must localize to lysosomes in order to induce perinuclear clustering because a PA-truncated RNF167 protein or a shorter isoform of RNF167 that does not localize to lysosomes did not induce the phenotype [80]. In another set of experiments, the overexpression of p50-Dynamitin, which disrupts the dynein–dynactin complex, and the inhibition of dynein with ciliobrevin both led to an increase in the peripheral distribution of lysosomes, suggesting that perinuclear clustering is dynein dependent [80]. In conditions promoting lysosomal exocytosis, RNF167-transfected cells display a substantial reduction in the surface expression of lysosomal-associated membrane protein 1 (Lamp1) when compared to controls, suggesting that RNF167 regulates lysosomal exocytosis [80]. In addition, RNF167 overexpression increased the amount of propidium iodide (PI) stained cells, signifying that the plasma membrane was neither intact nor repaired after streptolysin-O (SLO) treatment-induced pore formation in the plasma membrane [80].

### 4.4. Pathological Dysfunctions Associated with RNF167

#### Cancer

In addition to the identification of the RNF13 variants in tumor samples, some RNF167 variants were identified and characterized as well [27]. Similar to the results of RNF13, RNF167’s variants, located within the PA or RING domain, abrogated the ability to induce giant endosomes in HEK293T cells [27]. Also, the authors demonstrated that RNF167 PA domain variant V98G, when co-expressed with WT RNF167, inhibits the formation of giant endosomes, therefore suggesting that the RNF167 V98G variant acts as a dominant-negative protein [27]. In another study, the K97N mutation in the PA domain was the only variant that did not induce lysosomal perinuclear clustering while increasing the surface expression of Lamp1, supporting the idea that RNF167 K97N increases lysosomal exocytosis [80]. Furthermore, cells overexpressing RNF167 K97N that were treated with SLO and Ca^2+^ to induce pore formation before allowing membrane resealing had lower PI staining than with WT RNF167, suggesting that the variant enhanced plasma membrane repair [80]. Overall, these studies suggest that PA domain variants of RNF167 found in tumor samples are mislocalized and might act as dominant negatives.

In an early study, it was reported that RNF167 polyubiquitinates Tumor-Suppressing Subchromosomal Transferable Fragment cDNA (TSSC5), a tumor suppressor, to degrade it via the proteasome pathway [75]. In this specific study, ectopically expressed RNF167 delays the G1-to-S transition in HeLa cells [75]. While the exact function of RNF167 in TSSC5 regulation is far from being understood [75], another study implicates RNF167 in cancer through the mammalian target of rapamycin complex 1 (mTORC1) [81]. RNF167 polyubiquitinates the protein Cytosolic arginine sensor for mTORC1 subunit 1 (CASTOR1) with a K29-linked Ub chain, which inhibits mTORC1 activation upon arginine deprivation, to promote CASTOR1 proteasomal degradation [81]. Interestingly, phosphorylation of CASTOR1 by the serine/threonine kinase AKT1 enhances the interaction between CASTOR1 and RNF167 [81]. Additionally, cells ectopically expressing RNF167 became insensitive to the arginine-mediated mTORC1 activation due to CASTOR1 degradation [81]. The phosphomimetic variant of CASTOR1, which causes lower CASTOR1 protein abundance, did not inhibit mTORC1 activation, tumor growth, or cell proliferation. The variant also led to a lower survival rate, suggesting that RNF167-mediated degradation leads to enhanced mTORC1 activation, cell proliferation, and tumorigenesis [81]. In vivo, high expression of RNF167 in breast tumors correlates with poor survival in human epidermal growth factor receptor 2 positive (HER2+) breast cancer while lower CASTOR1 expression was correlated with poor survival in estrogen receptor-positive (ER+) breast cancer [81].

Altogether, these important studies provided some mechanistic insight regarding RNF167 dysfunction in cancer cells. However, these same studies highlight the importance of further investigating the function and the Ub ligase activity of tumor-associated RNF167 variants.

## 5. ZNRF4

Zinc and Ring Finger 4 (ZNRF4, also referred to as Nixin) was first identified in 1999 when Fujii et al. isolated genes whose expression was induced in mouse spermatogenic cells [82]. At the time, they called the protein spermatic-specific RING zinc finger (sperizin) [82]. Using northern blot analysis, mouse sperizin was exclusively found in testis. The human ZNRF4 sequence (Uniprot Accession Q8WWF5) includes the characteristic domains of the family (Figure 2).

The sperizin gene expression was detected in pre-pubertal testis beginning at 23 days old, increased at 29 days old, and then declined in adult testis (35 days old), suggesting that it may be involved in mouse spermatogenesis [82]. Interestingly, sperizin expression was increased following a retinol treatment in vitamin A-deficient rat testes [83]. Vitamin A is known to be critical in maintaining mammalian spermatogenesis and sperizin could be an important downstream protein of this cellular process [83]. Furthermore, to our knowledge, no study has specifically investigated the role of the human ZNRF4 protein in spermatogenesis, but it is worth noting that ZNRF4 RNA expression in humans appears to be restricted to the testis as well [61]. However, while single-nucleotide variations of ZNFR4 have been discovered in tumors, it should be noted that studies have yet to report the pathological impact of these variants.

### 5.1. Calnexin

While the overexpression of GFP-tagged sperizin (mouse protein) leads to a cytosolic localization in HeLa cells, the overexpression of ZNRF4-YFP (human protein) displays an ER localization in HeLa and HCT116 cells [82,84,85]. Accordingly, ZNRF4 is implicated in the degradation of calnexin, an ER-localized chaperone [84]. Importantly, the ectopic expression of WT, but not DN ZNRF4, reduces calnexin staining in HeLa cells [84]. The use of the proteasomal inhibitor MG132 demonstrated that ubiquitination of calnexin led to proteasomal degradation, confirming the important role that ZNRF4 plays in calnexin turnover [84].

### 5.2. RIP2

A recent human genome-wide RNAi screen was performed to find negative regulators of nucleotide-binding oligomerization domain 2 (NOD2)-induced nuclear factor-κB (NF-κB) activation in HEK293T cells [85]. The authors discovered that ZNRF4 knockdown led to an increased response of NOD2-dependent NF-κB activation. Conversely, the ectopic expression of ZNRF4 in HEK293T led to a reduction in NOD2-induced NF-κB activation in a dose-dependent manner [85]. Using a DN ZNRF4 variant as a negative control, the authors confirmed that the overexpression of WT ZNRF4 led to reduced NOD2-dependent NF-κB activation and identified receptor-interacting protein 2 (RIP2), a major signal integrator for the NOD1 and NOD2 pathways, as the substrate [85]. The treatment with MG132 rescued RIP2 degradation when ZNRF4 was overexpressed, indicating that ubiquitination of RIP2 by ZNRF4 leads to proteasomal degradation. Importantly, this is consistent with the fact that ZNRF4 promotes K48-linked ubiquitination of RIP2 [85]. Additionally, the ZNRF4 knockdown in MPD-treated human monocyte-derived macrophages increased the secretion of cytokines, such as interleukin-8 (IL-8), tumor necrosis factor α (TNF-α), and interleukin 1β (IL-1β) [85]. Research also demonstrated a role for ZNRF4 in NOD2-induced tolerance in vivo as ZNRF4 knockdown mice lost their ability to induce NOD2 tolerance when compared to WT mice [85]. Overall, these studies suggest that ZNRF4 has a function in various biological processes, including spermatogenesis, ER homeostasis and NOD2 signaling [82,83,84,85].

## 6. Conclusions and Future Perspectives

A lot of work has been done in the last 20 years to better understand the role of the human Godzilla homologues, RNF167, RNF13, and ZNRF4. Table 1 summarizes the biological function and pathological dysfunctions of these E3 ligases, as well as the known substrates and roles of their ubiquitination. However, many unanswered questions remain.

**Table 1:** Summary of the substrates, the role of their ubiquitination, and their known biological functions and pathological dysfunctions for each E3 ligase discussed in the current review.

E3 Ligase	Substrate	Role of Ubiquitination	Biological Function	Pathological Dysfunction
Godzilla	Syb [33,45]	Not determined (N.D.)	Regulation of recycling endosome trafficking [33]	N.D.
		N.D.	Wingless transcytosis [45]	N.D.
RNF13	N.D.	N.D.	Ectopic expression promotes spontaneous growth of neurites in PC12 cells [60]	N.D.
		N.D.	mRNA expression increases with dibuturyl-cAMP treatment in B35 cells [47]	N.D.
	Snapin [62]	K29-linked poly-Ub	SNARE complex assembly [62]	Mice display deficit in learning [62]
		increased expression [62]		
	N.D.	N.D.	Increased MMP-9 activity [49]	Cancer [49,66]
		N.D.	Regulation of GM-CSF concentration [66]	
		N.D.	Altered endolysosomal system [27,59]	Developmental and epileptic encephalopathy 73 [59]
				Cancer [27]
	IRE1α	N.D.	Regulation of ER stress [48]	Developmental and epileptic encephalopathy 73 [74]
	(Interacts but not shown to be a substrate) [48]			Mouse model of Parkinson’s disease [73]
				Atherosclerotic plaques [72]
	N.D.	N.D.	Regulation of muscle cell proliferation through regulation of IL-4 and IL-6 [65,68]	N.D.
RNF167	GluA2 [76,77]	Regulated surface expression [76]	Regulation of neuronal synaptic strength [76]	N.D.
	Vamp3 [33]	N.D.	Regulation of recycling endosomes [33]	N.D.
	Arl8B [78]	Degradation via the proteasome-dependent pathway [78]	Lysosomal positioning [78]	Cancer [75,80,81]
	N.D.	N.D.	Lysosomal exocytosis [80]	
	CASTOR1 [81]	K29-linked polyUb chain	Inhibition of mTORC1 activation [81]	
		Degradation via the proteasome-dependent pathway [81]		
	TSSC5 [75]	Degradation via the proteasome-dependent pathway [75]	Delays G1-to-S transition in HeLa cells [75]	
ZNRF4	N.D.	N.D.	Spermatogenesis in mice? [83]	N.D.
	Calnexin [84]	Degradation via the proteasome-dependent pathway [84]	ER homeostasis [84]	N.D.
	RIP2 [85]	K48-linked poly-Ub chain	Negatively regulates NOD2 signaling [85]	N.D.
		Degradation via the proteasome-dependent pathway [85]		

**Figure 3:**
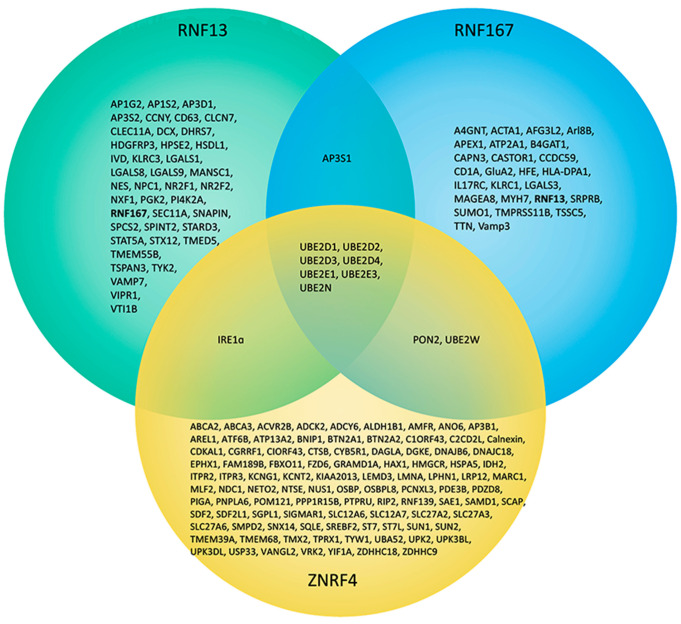
Venn diagram comparing the interactors of RNF13, RNF167, and ZNRF4. Interactors of RNF13 (green), RNF167 (blue), and ZNRF4 (yellow) discovered by both low and high throughput assays are displayed [33,48,54,55,56,59,62,75,76,77,78,81,84,85,87,90,91,92,93,94,95,96,97].

Over the last two decades, the use of high-throughput screening (HTS) has increased dramatically since it can repeatedly test thousands of samples per day [86]. For instance, Huttlin et al. expressed hundreds of FLAG-HA-tagged baits in HEK293T cells and combined immunopurification and mass spectrometry to identify interactors of these bait proteins [54]. As shown in Figure 3, multiple interactors for RNF167, RNF13, and ZNRF4 were found using either this method or a similar one. Interestingly, these Godzilla homologues all have in common interactions with the UBE2D family, UBE2E1, UBE2E3, and UBE2N, which can give information on the type of Ub linkage patterns these E3 ligases can perform (Figure 1) [75,77,87]. Although no specific molecular mechanisms can be revealed by using HTS, it can, however, offer a starting point by providing putative interactors. For example, while there is very limited information available for ZNRF4, other than the fact that its expression was linked to spermatogenesis in mice [82], it is still unclear whether this function applies to humans as well. Importantly, the discovery of Frizzled-6 (FZD6) as one of the interactors for ZNRF4 [55,56] is quite intriguing since FZD6 knockout mice show a severe reduction in sperm counts [88,89]. Therefore, studies are needed in order to determine the validity of a highly speculative mechanism where ZNRF4′s role in spermatogenesis may be through interaction or regulation of FZD6.

In contrast to the limited knowledge regarding the interactors of ZNRF4 and its biological function, more information is available for both RNF167 and RNF13. For instance, we now know that RNF167 ubiquitinates TSSC5 leading to its degradation [75]. How RNF167 regulates and controls the cell cycle, however, or the consequence of a dysfunction in this pathway is unknown. It is possible that RNF167 has substrates in diverse subcellular locations and needs to be trafficked depending on the cellular context. Accordingly, RNF167 substrates Vamp3, Arl8B, and GluA2 have all been characterized to transit within the endolysosomal pathway. This suggests that RNF167 traffics to the endolysosomes via a mechanism that is anticipated to be similar to the reported interaction between the canonical di-leucine motif of RNF13 and AP-3 [59], which is supported by the fact that both RNF13 and RNF167 have AP3S1 as common interactor (Figure 3) [55,56]. Thus, it is possible that the di-leucine motif is required for the interaction with the AP complexes, while the PA domain might be critical for the interaction with proteins that allow the proper endosomal targeting of both RNF13 and RNF167. An interaction was also identified between ZNRF4 and IRE1α, which was previously demonstrated to interact with RNF13 (Figure 3) [48,55,56]. Although their respective intracellular localization differs across various experimental settings, it may be of interest to study how these two E3 ligases regulate the same protein, and if some type of redundancy exists between them.

Finally, we cannot overlook the multiple studies that have shown how the specific domains are important for endosomal and lysosomal localization. For example, cancer-associated RNF167 variants K97N and V98G in the PA domain led to mislocalized proteins [27,80]. Furthermore, in order to better understand how RNF167 and RNF13 are regulated, we must determine the impact of PTMs, such as ubiquitination and phosphorylation (Table 2). To our knowledge, a comprehensive molecular investigation of PTMs on RNF13 and RNF167 has not been reported, although it is known that RNF13 and RNF167 are glycosylated and that RNF13 is trafficked to the nucleus following PKC activation by PMA treatment [50]. Therefore, it would be interesting to evaluate if PKC phosphorylates, either T309, S319, or T380, on RNF13 to induce nuclear localization or if PKC acts through an intermediate effector. Hopefully, future studies will provide clearer mechanisms as to how RNF167 and RNF13 reach the endolysosomal compartment and how their Ub ligase activities regulate the vesicular dynamics and the degradation of the plasma membrane cargo. This is critical since the list of pathologies associated with RNF167 and RNF13 is growing.

**Table 2:** Information on gene and post-translational modifications of human homologues of Godzilla.

Gene	Post-Translational Modifications
RNF167 ID 26001 Position 17p13.2	Ubiquitination: K97, K156, K210, K214, K223, K242, K266, K272 [98,99,100,101] Methylation: R277 [102] Phosphorylation: T288, S341, S345 [103] Glycosylation: N33, N79 [76]
RNF13 ID 11342 Position 3q25.1	Ubiquitination: K107, K224, K225, K230, K232, K233, K252, K265, K273, K275, K282 [98,99,100,101,104,105,106] Phosphorylation: T309, S319, T380 [103,107] Glycosylation: N70, N75, N88 [49,108]
ZNRF4 ID 148066 Position 19p13.3	Phosphorylation: T70 [109] Glycosylation: N107, N152, N229 [84]

## Data Availability

Not applicable.

## Abbreviations

AMPA	α-amino-3-hydroxy-5-methyl-4-isoxazolepropionic acid
AMPAR	AMPA receptor
AMPAR-EPSC	Evoked excitatory postsynaptic AMPAR currents
AP	Adaptor protein
Arl8B	ADP-ribosylation factor-like protein 8B
ASK1	Apoptosis signal-regulating kinase 1
C-RZF	Chicken RING zinc finger
CASTOR1	Cytosolic arginine sensor for mTORC1 subunit 1
CFM	Chicken fetal myoblasts
CHO	Chinese hamster ovary
CTX	Cardiotoxin
DEE73	Developmental and epileptic encephalopathy 73
DN	Dominant-negative
DUB	Deubiquitinase
E	Glutamic acid
E1	Ubiquitin-activating enzyme
E2	Ubiquitin-conjugating enzyme
E3	Ubiquitin ligase
EEA1	Early endosome antigen 1
EGFR	Epidermal growth factor receptor
ER	Endoplasmic reticulum
ER+	Estrogen receptor positive
FZD6	Frizzled-6
GM-CSF	Granulocyte macrophage colony-stimulating factor
HECT	Homology to E6AP Carboxyl Terminus
HEK293T	Human embryonic kidney 293
HER2+	Human epidermal growth factor receptor 2 positive
HTS	High-throughput screening
IL-1β	Interleukin 1β
IL-4	Interleukin-4
IL-6	Interleukin-6
IL-8	Interleukin-8
INM	Inner nuclear membrane
IRE1α	Inositol-requiring enzyme 1α
JNK	c-Jun N-terminal kinase
Lamp1	Lysosomal-associated membrane protein 1
M6P	Mannose-6-Phosphate
MARCH	Membrane-associated RING-CH
MMP-9	Matrix metallopeptidase 9
MPTP	1-methyl-4-phenyl-1,2.3.6-tetrahydropyridine
mTORC1	Mammalian target of rapamycin complex 1
NF-κB	Nuclear factor-κB
NLS	Nuclear localization signal
NOD2	Nucleotide-binding oligomerization domain 2
Ox-LDL	Oxidized low-density lipoprotein
P	Proline
PA-TM-RING	Protease-associated transmembrane RING
PA	Protease-associated
PDAC	Pancreatic ductal adenocarcinoma
PI	Propidium iodide
PKC	Protein kinase C
PMA	Phorbol 12-myristate 13-acetate
PTM	Post-translational modification
RBR	RING-Between-RING
RING	Really Interesting Novel Gene
RIP2	Receptor-interacting protein 2
RNF13	Ring Finger Protein 13
RNF167	Ring Finger Protein 167
S	Serine
shRNF13	Short hairpin RNA specific for RNF13
siRNA	Small interfering RNA
SLO	Streptolysin-O
SNAP-25	Synaptosome associated protein 25
Snapin	SNARE-associated protein
SNARE	Soluble N-ethylmaleimide-sensitive-factor Attachment protein Receptor
SNV	Single-nucleotide variations
SP	Signal peptide
Sperizin	Spermatic-specific ring zinc finger
STS	Staurosporine
Syb	Synaptobrevin
T	Threonine
TA	Tibialis anterior
TH-IR	Tyrosine hydroxylase-immunoreactive
TM	Transmembrane
TNF-α	Tumor necrosis factor α
TNF	Tumor necrosis factor
TRAF2	TNF receptor-associated factor 2
TRIM	Tripartite motif-containing
TSSC5	Tumor-Suppressing Subchromosomal Transferable Fragment cDNA
Ub	Ubiquitin
Vamp3	Vesicle-associated membrane protein 3
WT	Wild type
XBP1	X-Box Binding Protein 1
ZNRF4	Zinc and Ring Finger 4
